# Assessment of global phase uncertainty in case-control studies

**DOI:** 10.1186/1471-2156-10-54

**Published:** 2009-09-14

**Authors:** Hae-Won Uh, Jeanine J Houwing-Duistermaat, Hein Putter, Hans C van Houwelingen

**Affiliations:** 1 Department of Medical Statistics and Bioinformatics, Leiden University Medical Center, Leiden, The Netherlands

## Abstract

**Background:**

In haplotype-based candidate gene studies a problem is that the genotype data are unphased, which results in haplotype ambiguity. The  measure [1] quantifies haplotype predictability from genotype data. It is computed for each individual haplotype, and for a measure of global relative efficiency a minimum  value is suggested. Alternatively, we developed methods directly based on the information content of haplotype frequency estimates to obtain global relative efficiency measures:  and  based on A- and D-optimality, respectively. All three methods are designed for single populations; they can be applied in cases only, controls only or the whole data. Therefore they are not necessarily optimal for haplotype testing in case-control studies.

**Results:**

A new global relative efficiency measure  was derived to maximize power of a simple test statistic that compares haplotype frequencies in cases and controls. Application to real data showed that our proposed method  gave a clear and summarizing measure for the case-control study conducted. Additionally this measure might be used for selection of individuals, who have the highest potential for improving power by resolving phase ambiguity.

**Conclusion:**

Instead of using relative efficiency measure for cases only, controls only or their combined data, we link uncertainty measure to case-control studies directly. Hence, our global efficiency measure might be useful to assess whether data are informative or have enough power for estimation of a specific haplotype risk.

## Background

When assessing the relationship between haplotypes and a disease outcome, a problem is that haplotypes are not directly observed. The genotype data are unphased, which results in haplotype ambiguity. This missing phase information causes reduction of the power in haplotype case-control studies, and the results may be misleading. Our interest is in two types of analyses; namely global test statistics to compare haplotype frequency distributions between cases and controls, and testing effects of individual haplotypes [2]. An optimal measure to quantify the amount of available information is needed for better understanding of the results obtained. Our main aim therefore is to develop a global relative efficiency measure that is directly based on the test statistic of a case-control study.

In the planning stage of case-control association studies, haplotype-tagging SNPs are often selected to have maximal power based on the pilot study of the target population or using information drawn from the International HapMap (). For this purpose, Stram *et al*. [1] proposed  that quantifies predictability of the individual haplotype from genotype data. For a measure of global efficiency it was suggested to take the minimum  value. Alternatively, Uh *et al*. [3] developed multivariate methods directly based on the information content of haplotype frequency estimates. The global relative efficiency measures,  and , were defined as the ratio of observed information relative to the complete data information based on A- and D-optimality [4,5], respectively. Nicolae [6] also proposed an A-optimality based measure in a broader framework. The  measure reflects the average information of the parameters, and  value simply relates to one diagonal element of the observed information matrix [3]. In contrast, the  measure takes possible correlations between the parameters into consideration. These three measures (,  and ) can be used for choosing tagSNPs to maximize information content on haplotypes and to maximize the power of the planned study. In the context of case-control studies these three measures, which are designed for single populations, are not readily applicable for case-control association studies. Therefore we propose a new measure, , which is optimal for assessing global relative efficiency of case-control studies using haplotypes.

O'Hely and Slatkin [7] have addressed a similar issue and provided a ratio *R *based on non-centrality parameters using likelihood ratio statistics. Their methods are based on non-centrality parameters, hence closely related to the issue of sample size in a case-control study. In general, enlarging sample sizes improves the power of the study. However, we argue that increasing the number of cases and controls with the same corresponding LD structure has little influence on relative efficiency with respect to phase uncertainty; *i.e*., resolution of haplotype phase does not depend on the sample size. Here our new relative efficiency measure  can be of great assistance to check whether data are informative enough for haplotype case-control studies and the results are correctly interpreted. For low values of a relative efficiency measure the haplotype-based inferences should be interpreted with caution even when sample sizes are large.

When conducted studies appear to be not informative enough for haplotype analysis (low values of ), one might want to resolve the haplotype phase. In principle, it is possible to resolve phase uncertainty either by laboratory work which is still costly, or by additional genotyping of family members. However, is it worth while to make these efforts? Regarding cost-effectiveness, a forward selection procedure based on the  measure is proposed for pinpointing the individuals (cases or controls) who are most responsible for the loss of information due to haplotype uncertainty. These same individuals have the highest potential to increase the power of the case-control study by resolving haplotype phase.

We briefly describe our methods for single populations and proceed to derive methods for case-control data sets. We illustrate our methods with the Interleukin-1*β *Gene Cluster Data. All computational work has been done using the programming language R [8]. An R program is available at .

## Results

### Application to the Interleukin-1*β *Gene Cluster Data

The data consist of a random sample of 886 subjects (ages 55-65 years) from a population-based cohort, the Rotterdam study [9,10]. Two polymorphisms within Interleukin-1*β *Gene (IL1*β*) and one within the IL-1 receptor (IL1RN) were chosen for haplotype association with the occurrence of radiographic osteoarthritis (ROA) in the hip, knee and hand. After removing missing data, ROA data consist of 714 unrelated subjects: 61 cases and 653 controls for hip ROA. In Table 1 for the whole population, cases and controls, the haplotype frequency estimates are given which were obtained by THESIAS [11]. This software uses stochastic expectation maximization (EM) algorithm. Pairwise Linkage Disequilibrium (LD) in controls was observed for the first two SNPs (*D' *= 0.71 and *r*^2 ^= 0.09) and for the second and third SNPs (*D' *= 0.44 and *r*^2 ^= 0.13). The relatively low values of *r*^2 ^indicated that none of the markers can be considered redundant in an association study. Meulenbelt *et al *[10] found (suggestive) positive association of two haplotypes **112 **and **121 **with *hip ROA *(*p*_**112 **_= 0.0008 and *p*_**121 **_= 0.0002). The corresponding values of Stram's [1] were 77.4% and 85.6%, which are less than the recommended 90% [1]. The range of the  values per haplotype was from 57% to 92%. Note that these  values indicate relative efficiency only per haplotype for the whole data. Hence, this measure might not be adequate to assess the global efficiency for haplotype testing in case-control studies.

**Table 1:** Haplotype frequency estimates of hipROA data

	**Haplotype**	**Total**	**Cases**	**Controls**
1	111	0.36	0.25	0.37
2	112	0.08	0.15	0.07
3	121	0.16	0.27	0.15
4	122	0.16	0.18	0.16
5	211	0.20	0.12	0.21
6	212	0.02	0.03	0.02
7	221	0.02	0.01	0.03

Since our example data set was extremely unbalanced - 61 cases versus 653 controls and the set of cases may be too small to cover the haplotype structure completely, we generated the more balanced data set of 500 cases and 500 controls based on the real data set. To investigate the performance of global efficiency measures, 1,000 data sets were generated.

#### Global relative efficiency of the data

In Table 2 the four relative efficiency measures - min(), , , and  - are given in cases only, controls only, and in the case-control study setting using the real hip ROA data. While the minimum [1] was 77.9% in cases and 59.3% in controls, for the specific case-control study our power-related measure  = 82.3%. Bearing in mind that we are mostly interested in assessing the effect of a subset of two haplotypes **112 **and **121**, and that these two haplotypes were found significantly associated with hip ROA, we computed the corresponding . The informativeness increased to 92.6%.

**Table 2:** Global relative efficiency.

	**nr of individuals**			**per group (%)**			**Case-control study (%)**	
	**Total**	**ambiguous**	**%**	**min**()				
**hipROA**								
control	653	212	32.5	59.3	86.4	89.8	82.3	92.6
case	61	22	36.1	77.9	81.4	78.5		

**Simulated data**^1^								
control	500	174	34.8	63.7	85.4	79.8	83.2	93.3
case	500	181	36.2	53.9	88.6	77.3		

For each group min(),  and  values were given, and for a the case-control study  value was computed in terms of power of the global statistic T in (8). The subscript 2,3 indicates the relative efficiency of the haplotypes 112 and 121. 1Results from one simulated sample.

Since the high values of  and  in controls might reflect imbalance of data - case-control ratio was about 1/10, we generated 500 cases and 500 controls based on the real data. The 95% confidence intervals based on 1,000 simulations were:  ∈ (58.4, 65.5),  ∈ (85.4, 89.5),  ∈ (71.4, 76.8),  ∈ (83.0, 87.4) and  ∈ (91.7, 94.8).

#### Selection of informative individuals

Suppose phase ambiguity of haplotypes in our data set can be resolved by additional laboratory work or genotyping family members, the question arises which individual should be selected first. In Table 3, we grouped individuals with identical genotypes. The characters of the group identifiers denote the genotype at the SNPs, where 1 and 2 stand for homozygote 1/1 and 2/2, and H denotes a heterozygote. The individuals of this genotypic group 1HH can have compatible haplotypes of **111, 112, 121 **and **122**. When there is no phase ambiguity - for example due to Linkage Disequilibrium (LD), the number of compatible haplotypes will be two. The order of the group identifications are determined by the sum of the diagonal elements - the column "loss per genotype" - of the loss matrix ℒ_*i *_in (3). Note that this method is comparable to A-optimality measure, and it is used for relative efficiency measure in [12]. The highest labels (1HH in cases and H1H in controls) denote the group with highest loss, therefore potentially highest for information gain. The values of the last row - the row "loss per haplotype" - information loss per haplotype. These values relate to Strams's  in the following manner: for example for haplotype **111**,  ~ 1 - 3.00/17.52. the haplotype **121 **has the largest information loss. Within **121 **the individuals contributing the largest loss are the type 1HH. Selecting (or resolving) one individual in this group will change the table, and we repeat the procedure. Whether we should select cases first cannot be determined using Table 3.

**Table 3:** Selection strategy for the subset based on information without taking into account correlations between haplotype frequency estimates.

**hipROA data**	**genotype**	**nr of individuals**	**111**	**112**	**121**	**122**	**211**	**212**	**221**	**loss per genotype**	**total loss**
Cases *n *= 61	1HH	10	0.25	0.25	0.25	0.25	0	0	0	1.00	
	HHH	7	0	0.03	0.18	0.19	0.19	0.18	0.03	0.79	
	H1H	2	0.19	0.19	0	0	0.19	0.19	0	0.77	
	HH1	3	0.04	0	0.040	0	0.04	0	0.040	0.16	
	no ambiguity	39									
	loss per haplotype		3.00	3.07	3.85	3.83	1.85	1.62	0.31		17.52

Controls *n *= 653	H1H	28	0.21	0.21	0	0	0.21	0.21	0	0.83	
	HH1	46	0.18	0	0.18	0	0.18	0	0.18	0.72	
	1HH	91	0.12	0.12	0.12	0.12	0	0	0	0.49	
	HHH	47	0	0.04	0.06	0.10	0.10	0.06	0.04	0.40	
	no ambiguity	441									
	loss per haplotype		25.29	19.09	22.23	15.760	18.59	8.52	10.34		119.81

**Simulated data**	**genotype**	**nr of individuals**	**111**	**112**	**121**	**122**	**211**	**212**	**221**	**loss per genotype**	**total loss**

Cases *n *= 500	1HH	83	0.25	0.25	0.25	0.25	0	0	0	1.00	
	HHH	40	0	0.03	0.11	0.13	0.13	0.11	0.03	0.55	
	H1H	26	0.11	0.11	0	0	0.11	0.11	0	0.15	
	HH1	32	0.04	0	0.04	0	0.04	0	0.04	0.15	
	no ambiguity	319									
	loss per haplotype		24.80	24.94	26.26	26.07	9.36	7.17	2.52		121.12

Controls *n *= 500	H1H	25	0.23	0.23	0	0	0.23	0.23	0	0.93	
	HH1	36	0.21	0	0.21	0	0.21	0	0.21	0.83	
	HHH	43	0	0.05	0.06	0.11	0.11	0.06	0.05	0.44	
	1HH	70	0	0.11	0.11	0.11	0.11	0	0	0.42	
	no ambiguity	326
	loss per haplotype		20.68	15.23	17.65	11.89	17.86	8.61	9.55		101.47

The group identifiers denote the genotype at the SNPs, where 1 and 2 stand for homozygote 1/1 and 2/2, and H denotes a heterozygote. The order of the group identifications are determined by the sum of the diagonal elements - the column "loss per genotype" - of the loss matrix ℒ_*i *_in (3). Individuals with higher loss will results in higher information gain, when their ambiguity could be resolved. The values of the last row, "loss per haplotype", show information loss per haplotype. The simulated data set is the same sample data set as in Table 2.

**Figure 1:**
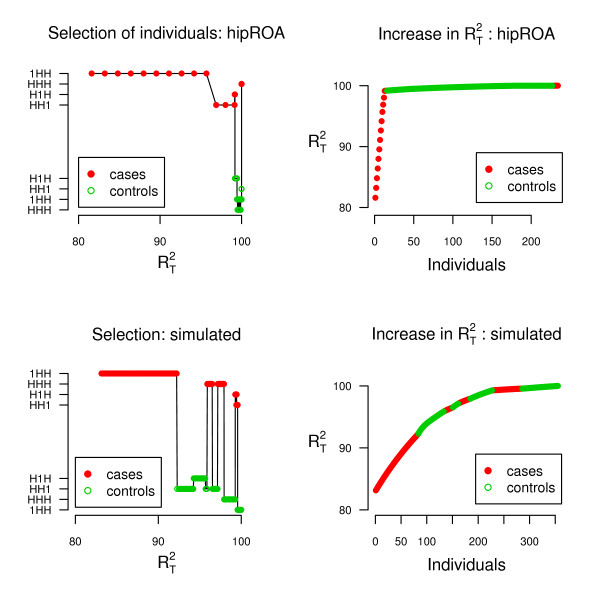
**Forward stepwise selection of informative individuals and the corresponding increase in  using real and simulated data**. To gain information efficiently forward stepwise selection of the most informative individuals is employed for maximizing the power of global test *T*, for the real hipROA data (upper panels: n(case) = 61 and n(control) = 653) and a comparable simulated data (lower panels: n(case) = n(control) = 500). (i) The left panels: The points represent the selection by . The groups in the *y*-labels are ordered as in Table 3: the upper part 1HH, HHH, H1H, HH1 represents the selection order for cases, and the lower part selection order H1H, HH1, 1HH, HHH for controls using the real data. Consequently, the jumps between the groups, and cases and controls are caused by using different methods. (ii) The right panels show the increase in  by resolving phase uncertainty.

Figure 1 shows the forward stepwise selection of individuals using  measure, specifically developed for case-control studies. The groups in the *y*-labels are ordered as in Table 3: the upper part 1HH, HHH, H1H, HH1 represents the selection order for cases, and the lower part selection order H1H, HH1, 1HH, HHH for controls using the real data. The points represent the selection by . At first, 10 case individuals with the type 1HH are chosen. Instead then selecting the HHH individuals who are the second in Table 3, a jump is made to HH1 individuals, and it indicates correlation between parameters. Hence, Figure 1 illustrates the discrepancies in using two different criteria. Especially the jumps between the groups, and cases and controls are caused by using different methods. In the real data, resolving case individuals increase information content dramatically. For comparison, results using the same simulated data set of 500 cases and controls based on real data as in Table 2 are given.

## Discussion

For case-control association studies using haplotypes it is of great importance to evaluate the data set whether it is appropriate to conduct haplotype-based analysis. This step enables us to interpret the results correctly. Therefore, we developed a global relative efficiency measure, , which was directly based on the test statistic of a case-control study. For testing a subset of haplotypes, ***s***, we proposed .

It has been noted that the extent of LD can be different between the case and control groups in a candidate region [13]. Our study also showed that the uncertainty of data clearly depends on the specific structure of data used. The  values were comparable using a unbalanced data set (the HipROA data) as well as using balanced simulated data sets which supposedly have the same structure as the real data. When the data are not informative enough to conduct haplotype-based analyses, say  ≤ 90%, tow options can be considered. One is to select individuals who have the highest potential to increase the power by resolving haplotypes, as discussed in the results section. The second is to make haplotype blocks [14] smaller until a pres-set  value is reached, whose limit would be the block containing a single SNP.

We did not address here which methods could be used to enlarge the efficiency of the study. It may be argued that the phase resolution by laboratory work is too costly. However, simply genotyping more individuals does not help in resolving phase ambiguity, assuming that additional cases and controls were selected from comparable populations as in the original data. For late-onset diseases it would not be possible to obtain samples of parents. However, in the planning stage of some studies, expected (remaining) information loss after genotyping parents could be calculated to make a balanced decision. In the same way, adding familial information from the sibling pairs could be an option. Putter *et al*. [12] showed that adding a sib increases information by 1/2 compared to adding parents, and adding the second sib by (1/2)^2^, the third sib by (1/2)^3 ^etc. That is, we need 4 or 5 sibs to obtain 90% of information by adding parents. Our methods are based on the assumption of Hardy-Weinberg equilibrium (HWE) in sample haplotype frequencies, in addition to a multiplicative model. Therefore, our relative efficiency measure would be influenced by the departure from HWE. As our *T*-statistic can be considered as a multi-allelic test, which is known to have inflated type 1 error rates when HWE is not satisfied [15,16]. Satten and Epstein [17] showed that the both prospective and retrospective approaches with a multiplicative model is robust to the HWE assumption in the target population. In the same paper, they also showed that the retrospective approach, which we used in our statistic, is superior to the prospective one. When the departure from HWE cannot be ignored, for example caused by inbreeding and population stratification, a variant of  based on retrospective likelihood can be developed using a fixation index.

## Conclusion

To assess the relative efficiency for haplotype testing in a case-control study, we developed methods based on the *T*-statistic as described in the Methods section. This measure indicates how much information is contained compared to the fully phased data for haplotype analysis in case-control studies. We also showed how this measure can be used for optimal selection of individuals who contribute most to information gain by resolving phase ambiguity.

By applying to the real data, we obtained the global relative efficiency  = 82.3% for haplotype analysis. Focusing on only two haplotype that are found significantly associated with disease, we obtained  = 92:6%.

## Methods

### Quantification of global relative efficiency in a sample

Suppose we have a sample of *n *unrelated individuals from a population. From each individual we observe *m *multilocus SNP-genotypes. Under Hardy-Weinberg equilibrium (HWE), the distribution of haplotypes is assumed to be multinomial, and the joint distribution of the paired haplotypes is equal to the product of the two marginal distributions. Here HWE assumption is required for haplotype distribution - and not for single SNPs - in the corresponding population. The haplotype will be described by a *k*(≤ 2^*m*^) dimensional vector *h *with its elements 0 or 1, and Pr(*h*_*j *_= 1) = *π*_*j *_denotes the frequency of haplotype *j *= 1,..., *k*, with . Note that each subject has two such haplotypes. We use the natural parametrization in *α *that is "symmetric" in the haplotypes [3,12]:



Note that the parameter vector *α *is not completely identifiable. We first derive all the formulas as if there is no constraint on *α*, and when necessary we transform them to the appropriate parameter space.

If there is no uncertainty, any (ordered) haplotype pair (*h*_1_, *h*_2_) of one individual may be described with a *k*-vector *H*_*j *_=  + , where *H*_*j *_∈ {0, 1, 2}, so-called haplotype dosage. Then, per subject, the log-likelihood *l*(***α***), the score function *U*(***α***) and the Fisher information *I*(***α***) are:



where



The total information based on *n *individuals is *I*_*comp *_= 2*nC*. The covariance matrix is given by

(1)

where (·^-^) denotes the Moore-Penrose generalized inverse [18].

In case of phase ambiguity, the haplotypes can be thought as (unphased) genotypes plus phase information. Hence, the complete data H can be partitioned as H = (G, Z), where G denotes the observed (incomplete) genotype data and Z the missing phase information. As Louis [19] observed the observed information can be expressed as *I*_G _= *I*_H _- *I*_H|G_. The loss, ℒ_*i *_= *I*_H|G;i_, caused by missing phase information for one individual *i *is then

(2)

where *f*_H|G _is the corresponding density. And, the observed information is given by

(3)

The corresponding covariance matrix of  is given by

(4)

The last expression is obtained by Taylor approximation given that  is small, and it shows that loss of information will cause increase in the covariance of estimates. When we have no ambiguities in the data, ℒ_*i *_equals to zero, and the covariance becomes simply *C*/(2*n*) in (1).

Note that the singular Fisher information *k *× *k *matrix (consequently the covariance matrix) can easily be transformed to the (*k *- 1) × (*k *- 1) matrix *I*. In Lehmann [20], it is described how an information matrix changes under reparametrization. Let a function *t *define as follows:



Then the matrix *J *contains the first partial derivatives of the function *t*,



*I *can be computed as *I *= *J*^T^I*(***α***)*J*. From now on, the Fisher information as well as covariance matrices are assumed to be properly transformed into an appropriate parameter space.

To assess global efficiency of data, the relative efficiency is defined by the ratio of information content of observed data to that of complete data. Let *I*_*obs *_as in (3) and *I*_*comp *_as in (1) denote observed information and complete information, respectively. Then based on A-optimality

(5)

where tr(*I*) is the trace of information matrix. To account for possible correlations between the parameter estimates, we propose  based on D-efficiency measure [21,22]. For *k *- 1 parameters, it is defined as

(6)

where |*I*| denotes the determinant of the matrix, and calculated as a product of nonzero eigenvalues. Note that this measure is invariant to transformation of parameters. High values of  and  indicate that data are informative to estimate haplotype frequencies.

Next, efficiency measure regarding a subset, ***s***, of the haplotype frequency estimates is considered. Partition the *k*-1 parameters as follows:



Treating ***π*_-*s *_**as a nuisance parameter *I*_***π ***_can be partitioned as

(7)

where ***I*_*s*, *s *_**is 2 × 2 matrix with respect to ***π*_*s*_**. The information content with respect to this subset ***s ***amounts to .

### Quantification of global relative efficiency in case-control studies

For a case-control study, we propose a new relative efficiency measure based on the power. Let  and  denote estimates of the frequencies of haplotype *j *= {1,..., *k *- 1} in controls and cases, respectively. The difference in haplotype frequencies is denoted as a vector . Then the global statistic is defined as follows:

(8)

which is χ^2 ^distributed with *k *- 1 degrees of freedom. For computation of global statistic *T*, the complete and observed covariance for cases and controls as in (1) and (4) can be plugged in the denominator of the statistic:  Then, the global relative efficiency concerning the power of T can be defined as follows:



where *T*_*obs *_and *T*_*comp *_denote observed and complete global statistic *T*, respectively. In case that the null hypothesis specifies only a subset of haplotypes and by treating the remaining haplotypes as a nuisance parameter, we use the classical score statistic when the null hypothesis is composite, as described in Cox and Hinkley [23]. Let Σ**_*s*, *s*_**, Σ**_*s*,-*s*_**, Σ**_-*s*, *s *_**and Σ**_-*s*,-*s *_**denote the corresponding subsets of the covariance matrix  in (8). As in (7), ***s ***denotes the subset of interest and ***π*_-*s *_**is considered as a nuisance parameter. Then, the global statistic concerning for the subset ***s ***is



and relative efficiency is denoted as 

In order to select the most informative individuals in a case control study, the forward stepwise selection procedure could be employed for maximizing the power of global test *T*; i.e., it is determined which multilocus combination of genotypes provides most information gain, when the phase ambiguity is resolved.

## Authors' contributions

H-WU performed the analyses and wrote the manuscript. All authors, H-WU, JJH-D, HP and JCvH, participated in the development of the methods, interpreted the results of the analysis, read the manuscript, and approved the final manuscript.

## References

[B1] Stram DO, Haiman JN, Hirschhorn JN, Altshuler D, Kolonel LN, Henderson BE, Pike ML (2003). Choosing haplotype-tagging SNPs based on unphased genotype data using a preliminary sample of unrelated subjects with an example from the multiethnic cohort study. Hum Hered.

[B2] Schaid DJ, Rowland CM, Tines DE, Jacobson RM, Poland GA (2002). Score tests for association between traits and haplotypes when linkage phase is ambiguous. Am J Hum Genet.

[B3] Uh HW, Houwing-Duistermaat JJ, Putter H, van Houwelingen JJC (2005). How to quantify information loss due to phase ambiguity in haplotype case-control studies. BMC Genet.

[B4] Atkinson AC, Donev AN (1992). Optimum Experimental Designs.

[B5] Fedorov V (1972). Theory of optimal experiments.

[B6] Nicolae DL (2006). Quantifying the amount of missing information in gentic association studies. Genet Epi.

[B7] O'Hely M, Slatkin M (2003). The loss of statistical power to distinguish population when certain samples are ambiguous. Theor Pop Biol.

[B8] The R project for statistical computing. http://www.r-project.org/.

[B9] Hofman A, Grobbee D, de Jong PT, Ouweland FA van den (1991). Determinants of disease and disability in elderly: the Rotterdam Elderly Study. Eur J Epi.

[B10] Meulenbelt I, Seymour AB, Nieuwland M, Huizinga TWJ, van Duijn CM, Slagboom PE (2004). Association of the Interleukin-1 gene cluster with radiographic signs of osteoarthritis of the hip. Arthritis & Rheumatism.

[B11] Tregouet DA, S E, Tiret L, Mallet A, Golmard JL (2003). A new maximum likelihood algorithm for haplotype-based association analysis: the SEM algorithm. Ann Hum Genet.

[B12] Putter H, Meulenbelt I, van Houwelingen JJC (2007). Relative efficiency of haplotype frequency estimation in sibships and nuclear families compared to unrelated individuals. Hum Hered.

[B13] Zaykin DV, Meng Z, Ehm MG (2006). Contrasting linkage-disequilibrium patterns between cases and controls as a novel association-mapping method. Am J Hum Genet.

[B14] van Minkelen R, de Visser MC, Houwing-Duistermaat JJ, Vos HL, Bertina RM, Rosendaal FR (2007). Haplotypes of IL1B, IL1RN, and IL1R2 and the risk of venous thrombosis. Arterioscler Thromb Vasc Biol.

[B15] Zheng G (2008). Can the allelic test be retired from analysis of case-control association studies?. Ann Hum Genet.

[B16] Sasieni PD (1997). From genotypes to genes: doubling the sample size. Biometrics.

[B17] Satten GA, Epstein MP (2004). Comparison of prospective and retreospective methods for haplotype inference in case-control studies. Genet Epi.

[B18] Rao CR, Mitra SK (1971). Generalized Inverse of Matrices and Its Applications.

[B19] Louis TA (1982). Finding the observed information matrix when using the EM algorithm. J R Stat Soc.

[B20] Lehmann EL (1983). Theory of point estimation.

[B21] Minkin S (1987). Optimal Designs for Binary Data. J Amer Stat Assoc.

[B22] Heise MA, Myers RH (1996). Optimal Designs for Bivariate Logistic Regression. Biometrics.

[B23] Cox DR, Hinkley DV (1974). Theoretical Statistics.

